# Are surgical and non-operating room intervention safe in the COVID-19 pandemic? A retrospective study

**DOI:** 10.1017/S0950268821002119

**Published:** 2021-09-16

**Authors:** Serap Aktas Yildirim, Zeynep Tugce Sarikaya, Halim Ulugol, Sanem Ozata, Ugur Aksu, Fevzi Toraman, Bulent Gucyetmez, Muzeyyen Iyigun, Ufuk Topuz, Mefkur Bakan, Dilek Altun, Huseyin Macika, Mehtap Selcuk, Emre Sahillioğlu, Muharrem Kocyigit, Emir Kilinc, Seher Kiran, Merve Seker, Ayse Sesin Kocagoz, Caglar Cuhadaroglu, Efe Onganer, Korhan Zakiroglu, Cem Alhan

**Affiliations:** 1 Department of Anesthesiology and Reanimation, Acibadem Mehmet Ali Aydinlar University School of Medicine, Istanbul, Turkey; 2 Department of Anesthesiology, Acibadem Atakent Hospital, Istanbul, Turkey; 3 Department of Biology, Faculty of Science, University of Istanbul, Istanbul, Turkey

**Keywords:** COVID-19 transmission, COVID-19, intensive care requirement, mortality, non-operating room intervention, surgery

## Abstract

Little is known about the impact of COVID-19 on the outcomes of patients undergoing surgery and intervention. This study was conducted between 20 March and 20 May 2020 in six hospitals in Istanbul, and aimed to investigate the effects of surgery and intervention on COVID-19 disease progression, intensive care (ICU) need, mortality and virus transmission to patients and healthcare workers. Patients were examined in three groups: group I underwent emergency surgery, group II had an emergency non-operating room intervention, and group III received inpatient COVID-19 treatment but did not have surgery or undergo intervention. Mortality rates, mechanical ventilation needs and rates of admission to the ICU were compared between the three groups. During this period, patient and healthcare worker transmissions were recorded. In total, 1273 surgical, 476 non-operating room intervention patients and 1884 COVID-19 inpatients were examined. The rate of ICU requirement among patients who had surgery was nearly twice that for inpatients and intervention patients, but there was no difference in mortality between the groups. The overall mortality rates were 2.3% in surgical patients, 3.3% in intervention patients and 3% in inpatients. COVID-19 polymerase chain reaction positivity among hospital workers was 2.4%. Only 3.3% of infected frontline healthcare workers were anaesthesiologists. No deaths occurred among infected healthcare workers. We conclude that emergency surgery and non-operating room interventions during the pandemic period do not increase postoperative mortality and can be performed with low transmission rates.

## Introduction

On 11 March 2020, the outbreak of severe acute respiratory syndrome coronavirus 2 (SARS-CoV-2) was declared a pandemic by the World Health Organisation due to the high transmissibility of the virus and the rapid increase in the number of cases [1]. Although presenting with heterogeneity according to age and comorbidities, the increase in the case fatality rate to *c*.4.5% proved to be a major concern for healthcare systems worldwide [2], and in many countries, the collapse of primary services has had serious repercussions due to the large increase in hospitalisations related to COVID-19. As a consequence, hospital systems have had to reorganise staff usage and bed resources, cancel elective surgery and only address emergency cases [3].

Some guidelines recommend symptom screening and polymerase chain reaction (PCR) testing before non-emergency procedures, but there are insufficient data on how to manage emergency cases [4]. Due to the variable sensitivity of tests and high levels of asymptomatic carriers, it is sometimes difficult to detect SARS-CoV-2 infection in some patients [5–7], and consequently, patients undergoing surgery may be at high risk for adverse postoperative outcomes [8]. Limited studies have shown that patients infected with SARS-CoV-2 have higher perioperative mortality and morbidity because of rapid acute respiratory distress syndrome progression and cardiovascular failure [9–11]. As surgical patients often have underlying comorbidities, surgical-induced immunosuppression in addition to COVID-19 infection can lead to an increased risk of mortality [12]. Likewise, healthcare workers are at risk of exposure to possibly high viral loads due to close contact with both unrecognised and confirmed cases [13], and therefore constitute a risk of both infecting themselves and other patients.

Despite the risks, some patients with diagnosed or unrecognised COVID-19 infections will require urgent surgery or interventions, and in the absence of necessary healthcare may become more vulnerable to COVID-19 infection. Indeed, cancellation or postponement of treatment may cause more harm to patients than the impact of SARS-CoV-2 in the long term [14–16]. For these reasons, it is necessary to gain an understanding of the safety of emergency surgery and intervention applications for both healthcare professionals and patients in pandemic hospitals.

As a healthcare group of six hospitals that treats COVID-19 patients as well as performing emergency surgery and non-surgical interventions, we conducted a retrospective study to determine how COVID-19 infection affected the outcomes of patients who underwent surgery and interventions during the first peak period of the pandemic. At the same time, we aimed to determine the risk of bidirectional transmission between healthcare workers and patients.

## Methods

### Overview

The primary objective of the study was to determine the effect of surgery and intervention on COVID-19 disease progression, ICU need and mortality; the secondary aim sought to evaluate the risk of virus transmission between patients and healthcare workers in this setting.

This retrospective study was conducted between 20 March and 20 May 2020 in six hospitals in Istanbul that belong to the Acibadem Health Group and Acibadem MAA University. Ethical approval was obtained from the Regional Ethical Committee of Acibadem MAA University (protocol no: ATADEK-2020/08).

Each of the six hospitals ceased elective surgery after they were declared pandemic hospitals by the Ministry of Health as of 20 March 2020 and from then only emergency surgery and non-operating room interventions were carried out. In light of the information in the literature, we made the following preparations to become a pandemic hospital in our healthcare group [17]. Operating rooms with at least one negative-pressure room for case inputs and outputs were allocated for COVID-19 cases. In all confirmed or suspected cases, personal protective equipment (PPE) and special intubation boxes were used for aerosol-generating procedures. At the first peak of the pandemic, due to the insufficiency of the SARS-CoV-2 PCR test kit (Bio-speedy SARS-CoV-2 rRT-PCR kit, Bioeksen, Turkey) used, only patients who were clinically suspicious or symptomatic (fever, muscle pain, fatigue, cough, shortness of breath, sore throat, diarrhoea, chest pain, anosmia or loss of taste) were tested [18]. A diagnosis of COVID-19 was made based on PCR positivity, chest computerised tomography (CT) results and/or the presence of clinical symptoms. Healthcare workers with symptoms of COVID-19, regardless of whether a PCR test or CT was performed, were isolated for 14 days to limit the exposure of healthy individuals.

### Patients

Patients who underwent emergency surgery or non-operating room intervention were included in the study. Healthcare workers and all COVID-19-positive inpatients who did not have surgery or intervention in the same period were included. Subjects under 18 years of age were excluded.

### Trial procedures

Patients were examined in three groups: group I consisted of those admitted for emergency surgery, group II of patients admitted for non-operating room interventions, and group III of inpatients with confirmed COVID-19 infection who did not undergo any surgery or intervention. Accompanying comorbidities; epidemiological, clinical, laboratory and radiological data; COVID-19 suggestive symptoms; PCR test results; anaesthesia management data; surgical type; and intensive care and mechanical ventilation needs were recorded for all COVID-19 patients, supportive treatment and antiviral therapy with hydroxychloroquine were initiated in the preoperative period. Unplanned intensive care unit (ICU) need was defined as admission to ICU (not related to surgery or intervention) within the first 10 days after surgery or an intervention in non-ICU patients. Patients with COVID-19 started treatment in the postoperative period and were accepted as in-hospital transmission cases since it was not known whether they had been admitted for their procedures in asymptomatic or presymptomatic periods.

### Statistical analysis

All data were analysed using RStudio Cloud 3.5.2 Eggshell Igloo. The Shapiro Wilk Normality Test was used to detect normal distributions. Data were presented as percentages and medians (minimum-maximum); categorical variables were compared by a *χ*^2^ test, and numerical variables by the Mann–Whitney *U* test. A Wilcoxon signed-rank paired test was used to compare preoperative and postoperative laboratory data in COVID-19 patient subgroups; a *P*-value of <0.05 was considered statistically significant.

## Results

### Clinical characteristics of the patients

In total, 1273 surgical (group I), 476 intervention (group II) and 1884 inpatients group III with a diagnosis of COVID-19 were examined. American Society of Anesthiologists (ASA) scores were significantly higher in group I and group II patients than group III (*P* < 0.1). Likewise, the COVID-19-positive group I and group II patients were more often classified as ASA class 2 or above than in group III (27.4% *vs.* 1.9%; *P* < 0.001). No significant difference was found between the mean age of COVID-19-positive group I and group II patients and the mean age of group III patients (56 (45–66) *vs.* 52 (38–67), *P* = 0.231).

Compared to group III patients, group I and group II with COVID-19 were more likely to have underlying hypertension (39.7% *vs.* 10.9%, *P* < 0.001), coronary artery disease (19.2% *vs.* 4.1%, *P* < 0.001), chronic obstructive pulmonary disease (16.4% *vs.* 2.0%, *P* < 0.001) and use of immunosuppressive drugs (60.3% *vs.* 0.2%, *P* < 0.001). Patient characteristics and demographic data are shown in Tables 1 and 2. The most common emergency surgery procedure was for Caesarean section (33.8%), and gastrointestinal endoscopy (49.8%) as an intervention. General anaesthesia was applied to 64.4% of surgical patients, while all interventions were performed with sedoanalgesia. The types of surgery and intervention, and anaesthesia management are shown in Table 3.

**Table 1.** Patient characteristics

	Group I (*n* = 1273)	Group II (*n* = 476)	Total (group I + group II) (*n* = 1749)	Group III (*n* = 1884)	*P*_1_	*P*_2_	*P*_3_
Age, years	37 (32–50)	47 (36–60)	39 (32–55)	52 (38–67)	***<0.001***	***<0.001***	***<0.001***
Male, *n* (%)	357 (28.0)	193 (40.6)	550 (31.4)	1036 (54.9)	***<0.001***	***<0.001***	***<0.001***
ASA classification > 2, *n* (%)	115 (9.0)	61 (12.8)	176 (10.1)	35 (1.9)	***<0.001***	***<0.001***	***<0.001***
Comorbidities, *n* (%)							
Hypertension	201 (15.8)	140 (29.4)	341 (19.5)	206 (10.9)	***<0.001***	***<0.001***	***<0.001***
DM	110 (8.6)	56 (11.8)	166 (9.5)	114 (6.1)	***0.006***	***<0.001***	***<0.001***
CRF	26 (2.0)	11 (2.3)	37 (2.1)	32 (1.7)	0.482	0.372	0.359
CAD	77 (6.0)	66 (13.9)	143 (8.2)	77 (4.1)	***0.012***	***<0.001***	***<0.001***
Immunosuppressive drug	81 (6.4)	94 (19.8)	175 (10.0)	3 (0.2)	***<0.001***	***<0.001***	***<0.001***
COPD	32 (2.5)	42 (8.8)	74 (4.2)	37 (2.0)	0.302	***<0.001***	***<0.001***
Smoking	218 (17.1)	92 (19.3)	310 (17.7)	318 (16.9)	0.864	0.208	0.506
CVA	18 (1.4)	10 (2.1)	28 (1.6)	18 (1.0)	0.235	***0.039***	0.082
COVID (+) patients, *n* (%)	43 (3.4)	30 (6.3)	73 (4.2)	1884 (100)	**<0.001**	***<0.001***	***<0.001***

CAD, coronary arterial disease; COPD, chronic obstructive pulmonary disease; CRF, chronic renal failure; CVA, cerebrovascular accident; DM, diabetes mellitus; group III, hospitalised COVID patients; group II, intervention patients; group I, surgical patients; *P*_1_, group I and group III; *P*_2_, group II and group III; *P*_3_, total (group I + group II) and group III.

**Table 2.** Patients characteristics and outcomes in patients with COVID-19

	COVID (+) group I (*n* = 43)	COVID (+) group II (*n* = 30)	Total (COVID (+) group I + group II) (*n* = 73)	Group III (*n* = 1884)	*P*_1_	*P*_2_	*P*_3_
Age, years	55 (43–66)	50 (10–65)	56 (45–66)	52 (38–67)	0.832	0.103	0.231
Male, *n* (%)	28 (66.7)	16 (53.3)	44 (61.1)	1035 (54.9)	0.132	1.000	0.303
ASA classification > 2, *n* (%)	7 (16.3)	13 (43.3)	20 (27.4)	35 (1.9)	***<0.001***	***<0.001***	***<0.001***
Comorbidities, *n* (%)							
Hypertension	12 (27.9)	17 (56.7)	29 (39.7)	206 (10.9)	***<0.001***	***<0.001***	***<0.001***
DM	4 (9.3)	6 (20.0)	10 (13.7)	114 (6.1)	0.353	***0.006***	***0.007***
CRF	0 (0.0)	3 (10.0)	3 (4.1)	32 (1.7)	0.394	***0.007***	0.121
CAD	4 (9.3)	10 (33.3)	14 (19.2)	77 (4.1)	0.083	***0.001***	***<0.001***
Immunosuppressive drug	20 (46.5)	24 (80.0)	44 (60.3)	3 (0.2)	***<0.001***	***<0.001***	***<0.001***
COPD	4 (9.3)	8 (26.7)	12 (16.4)	37 (2.0)	***<0.001***	***<0.001***	***<0.001***
Smoking	16 (37.2)	5 (16.7)	21 (28.8)	318 (16.9)	***<0.001***	1.000	***0.007***
CVA	0 (0.0)	2 (6.7)	2 (2.8)	18 (0.1)	0.525	***0.032***	0.131
Preoperative COVID (+) patients, *n* (%)	39 (90.7)	28 (93.3)	67 (88.9)				
ICU requirement, *n* (%)	18 (41.9)	5 (17.9)	23 (35.9)	–	–	–	–
MV requirement, *n* (%)	10 (55.6)	3 (60.0)	13 (56.5)				
MV duration, days	16 (12–24)	13 (2–28)	14 (10–27)				
LOS-ICU, days	19 (10–22)	15 (2–26)	17 (8–26)				
LOS-hospital, days	26 (22–32)	19 (17–30)	22 (20–31)				
Mortality	1 (2.6)	1 (3.6)	2 (3.1)				
Postoperative COVID (+) patients, *n* (%)	4 (9.3)	2 (6.7)	6 (8.2)	–	–	–	–
ICU requirement, *n* (%)	1 (25.0)	1 (50.0)	2 (33.3)				
MV requirement, *n* (%)	1 (25.0)	1 (50.0)	2 (33.3)				
Mortality	0 (0.0)	(0.0)	(0.0)				
ICU requirement, *n* (%)	19 (44.2)	6 (20.0)	25 (31.5)	460 (24.4)	***0.033***	0.443	***0.044***
Mortality, *n* (%)	1 (2.3)	1 (3.3)	2 (2.7)	57 (3.0)	0.809	1.000	0.904

CAD, coronary arterial disease; COPD, chronic obstructive pulmonary disease; CRF, chronic renal failure; CVA, cerebrovascular accident; DM, diabetes mellitus; group III, hospitalised COVID patients; ICU, intensive care unit; group II, intervention patients; group I, surgical patients; *P*_1_, group I and group III; *P*_2_, group II and group III; *P*_3_, total and group III.

**Table 3.** Types of surgery and intervention

Surgeries, *n* (%)	1273
Procedures	
Caesarean section	430 (33.8)
Gynaecologic oncology surgery	212 (16.7)
Abdominal surgery	164 (12.9)
Orthopaedic surgery	148 (11.6)
Urologic oncology surgery	107 (8.4)
Cardiovascular surgery	56 (4.4)
Neurosurgery	51 (4.0)
ENT surgery	51 (4.0)
Thoracic surgery	20 (1.6)
Plastic and reconstructive surgery	18 (1.4)
Retroperitoneal surgery	8 (0.6)
Ophthalmic surgery	8 (0.6)
Anaesthesia managements	
General	820 (64.4)
Regional	396 (31.1)
CSEA in C/S	327 (82.6)
Non-operating room interventions, *n* (%)	476
Procedures	
Endoscopy	237 (49.8)
Normal vaginal delivery	82 (17.2)
Interventional radiology	20 (4.2)
Radiation therapy	16 (3.4)
*In vitro* fertilisation	13 (2.7)
MRI	12 (2.5)
Brachytherapy	5 (1.1)

ENT, ear nose and throat; CSEA, combined spinal-epidural anaesthesia; C/S, caesarean section; MRI, magnetic resonance imaging.

### COVID-19 PCR test results

Table 4 shows that of the 232 patients in group I (preoperative period) tested by PCR, 15 (6.5%) were positive. Similarly, in group II patients, of 110 (23.1%) screened, only two yielded positive results. Overall, the prevalence of COVID-19 during the preoperative period was 3.1% and 5.9% in groups I and II, respectively, while in the postoperative period, the corresponding frequencies were 0.3% and 0.4%. Almost all (92%) of group III patients were screened, with 12.8% positivity; and the great majority of this group (87.2%) were diagnosed with COVID-19 based on thorax CT results or symptomology. PCR and symptoms data for all patients in the perioperative period are shown in Table 4, and corresponding laboratory data in Supplementary Table.

**Table 4.** Patient PCR data, symptoms and prevalence

	Group I (*n* = 1273)	Group II (*n* = 476)
*Preoperative period*		
The number of PCR test, *n* (%)		
Total	232 (18.2)	110 (23.1)
Positive	15 (6.5)	2 (1.8)
The number of CT, *n* (%)		
Total	151 (11.9)	78 (16.4)
Positive infiltration	34 (22.5)	26 (30.8)
Patients who have only clinical symptoms, *n* (%)	4 (0.3)	0 (0.2)
Treated patients, *n* (%)	39 (3.1)	28 (5.9)
Prevalence (%)	3.1	5.9
Symptoms, *n* (%)		
Cough	18 (46.2)	7 (28.0)
Fever	14 (35.9)	5 (20.0)
Arthralgia	8 (20.5)	3 (12.0)
Throat ache	3 (7.7)	1 (4.0)
Neurological findings	2 (5.1)	1 (4.0)
Diarrhoea	2 (5.1)	1 (4.0)
Anosmia	1 (2.6)	0 (0.0)
Postoperative period		
	Group I (*n* = 1273)	Group II (*n* = 476)
The number of PCR test, *n* (%)		
Total	47 (3.7)	18 (3.8)
Positive	2 (4.3)	2 (11.1)
The number of CT, *n* (%)		
Total	19 (3.0)	18 (4.2)
Positive infiltration	3 (15.8)	1 (5.6)
Patients who have only clinical symptoms, *n* (%)	1 (0.1)	0 (0.0)
Treated patients, *n* (%)	4 (0.3)	2 (0.4)
Prevalence (%)	0.3	0.4
Symptoms, *n* (%)		
Cough	3 (75.0)	2 (100.0)
Fever	2 (50.0)	1 (50.0)
Arthralgia	1 (25.0)	1 (50.0)
Throat ache	0 (0.0)	0 (0.0)
Neurological findings	0 (0.0)	0 (0.0)
Diarrhoea	0 (0.0)	0 (0.0)
Anosmia	0 (0.0)	0 (0.0)

Group II, intervention patients; group I, surgical patients; CT, computed tomography; PCR, polymerase chain reaction.

### ICU need and mortality

ICU admittance for COVID-19-positive patients was required for 19 (44.2%) of group I patients, 20% of group II and 24.4% of group III (Table 4). When COVID-19-positive and negative patients were compared, positive group I patients required ICU admission more frequently than negative patients (46.2% *vs.* 2.3%, *P* < 0.001). Similarly, positive group II patients needed more intensive care than negative patients (20% *vs.* 2%, *P* < 0.001). However, there was no significant difference in their mortality rates (*P*1 = 0.805 *vs. P*2 = 0.898). The overall mortality rate was 2.3% in COVID-19-positive group I patients, 3.3% in positive group II subjects and 3% in group III.

Comparison of COVID (+) surgical and intervention patients' ICU requirement is shown in Table 5.

**Table 5.** Comparison of COVID (+) surgical and intervention patients' ICU requirement

	ICU requirement (−) (*n* = 48)	ICU requirement (+) (*n* = 25)	*P*
Age, years	52 ± 17	59 ± 14	0.088
Male, *n* (%)	28 (58.3)	12 (48.0)	0.400
ASA classification > 2, *n* (%)	15 (31.3)	18 (72.0)	***<0***.***001***
Comorbidities, *n* (%)			
Hypertension	25 (52.1)	12 (48.0)	0.741
DM	7 (14.6)	4 (16.0)	0.872
CRF	5 (10.4)	1 (4.0)	0.344
CAD	9 (18.8)	5 (20.0)	0.898
Immunosuppressive drug	9 (18.8)	9 (36.0)	0.105
COPD	9 (18.8)	7 (28.0)	0.365
Smoking	12 (25.0)	7 (28.0)	0.782
CVA	2 (4.2)	3 (12.0)	0.209

CAD, coronary arterial disease; COPD, chronic obstructive pulmonary disease; CRF, chronic renal failure; CVA, cerebrovascular accident; DM, diabetes mellitus.

### Infected patients and healthcare workers

In our 968-bed healthcare group, a total of 5874 healthcare workers, 20.3% of whom were doctors, 39% of whom were auxiliary staff, and 40.7% of whom were nurses and technicians, worked during the pandemic period. A total of 73 anaesthesiologists worked during the pandemic in our six hospitals, and only three of them were infected with SARS-CoV-2.

A total of 9889 PCR tests were performed in hospitals, with a positivity rate of 12.8%. Hospital staff received a total of 1824 tests, with a positivity rate of 7.8%. No deaths occurred among infected healthcare workers. In this period, the prevalence of COVID-19 PCR positivity among hospital workers was 2.4%.

Four patients in group I and two in group II had positive PCR test results after surgery and intervention and were presumed to represent in-hospital contamination.

The number of contaminations and the disease prevalence among the healthcare workers and infected patients are shown in Table 6.

**Table 6.** Infected patients and healthcare workers ratios

	Group I (*n* = 1273)	Group II (*n* = 476)	Group III (*n* = 1884)
Infected patients, *n* (%)	4 (0.3)	2 (0.4)	–
Infected frontline healthcare workers, *n* (%)	17 (1.0)		72 (3.8)
Anaesthesiologist, *n* (%)	3 (0.2)		
Anaesthesia technician, *n* (%)	2 (0.1)		–
Surgeon, *n* (%)	6 (0.5)	–	–
Doctor, *n* (%)	–	4 (0.8)	42 (2.2)
Nurse, *n* (%)	1 (0.1)	1 (0.2)	30 (1.6)
COVID-19 prevalence for hospital healthcare workers, (%)	2.4		

Group III, hospitalised COVID patients; group II, intervention patients; group I, surgical patients.

## Discussion

SARS-CoV-2 is highly contagious and is primarily transmitted through breath aerosols and skin contact. Consequently, both healthcare workers and patients are at risk during surgery and interventions [19, 20]. This has become a major problem, especially for institutions that treat COVID-19-positive patients and follow up on other patients at the same time. The concern that COVID-19 could adversely affect the prognosis of patients who undergo surgery has also created uncertainty about which patients should be allowed to have surgery.

The results of this study show that COVID-19 poses a significant risk for patients undergoing emergency surgery or intervention in terms of the need for intensive care. Nevertheless, we found that mortality did not increase significantly compared to patients treated in the dedicated COVID-19 ward over the same period. Limited data are available in the literature to explain patient outcomes but the consensus appears to be that postoperative mortality rates with COVID-19 have increased in different patient groups [9, 21]. However, to the best of our knowledge, there are no studies on the postoperative prognosis of COVID-19 patients who undergo a non-operating room intervention. In some publications, increased postoperative mortality after surgery has been demonstrated in COVID-19 patients overall, but comparisons with COVID-19 inpatients who did not undergo surgery and interventions in the same period have not been made [11].

Patients with COVID-19 who underwent surgery were notable for their advanced age, male gender, hypertension and use of immunosuppressive drugs compared with non-infected patients. Although the need of such patients for postoperative intensive care was 20 times higher than for infection-negative counterparts, there was no significant difference in mortality (*P* = 0.805). In our view, the secondary impact created by surgery increased the need for ICU admittance in our patients, but that antiviral treatment initiated before surgery may have had a positive effect on mortality-related COVID-19 infection.

We found a similar relationship in the mortality rate for preoperatively COVID-19-diagnosed intervention patients, but they had fewer ICU needs than the surgical group. Intervention patients did not experience any secondary trauma caused by surgery as surgical patients did, and all patients received sedoanalgesia throughout their procedures. However, while the intervention patients had higher rates of pneumonia and immunosuppressive drug use, they required ICU admittance less frequently than surgical patients. Patients who have undergone surgery are susceptible to postoperative complications and infections due to surgery-induced immunosuppression, mechanical ventilation performed during general anaesthesia and existing comorbidities [12]. Therefore, intervention patients are at an advantage in terms of postoperative complications since they are not exposed to general anaesthesia and surgical trauma. We found that interventions performed with sedoanalgesia that did not require endotracheal intubation and mechanical ventilation in COVID-19 patients do not increase the need for intensive care and mortality which suggests that interventions requiring sedoanalgesia can be performed more safely than surgery in patients with COVID-19. The guidelines published by the American College of Surgeons in March 2020 [22], when the pandemic was at its peak, recommended the implementation of non-surgical treatments to delay or prevent the need for surgery and postpone non-critical procedures. COVID-19-positive intervention and surgical patients who needed intensive care had higher ASA scores. It follows that it is necessary to be more selective when deciding on surgery and intervention in high-risk patients. If intervention is safer than surgery for such patients, it can be applied more safely as an alternative measure, especially in patients at risk of having surgery during other peaks of the pandemic [22].

In a series of 34 patients from China, COVID-19 patients following surgery reported a need for ICU admittance 44% of the time; the perioperative mortality rate was 21%, and all 34 patients had pneumonia [9]. In a comparative series of 36 patients in an American study, 50% of patients had pneumonia, while 36% were admitted to the ICU; the mortality rate was found to be 17% [11]. In our case series, while 31% of 73 COVID-19 patients required ICU admittance, with a mortality rate of only 2.7%. Among our patients, 82% showed evidence of pneumonic infiltration on a thorax CT, 58% underwent surgery and the remainder had an intervention that did not require intubation and mechanical ventilation which might account for the lower mortality. In addition, estimation of mortality rates is most likely complicated by factors such as different case definitions and testing strategies in different countries, variations in treatment applied at different stages of the disease, and the quality of patient care. Although its efficacy was not proven in all our preoperatively diagnosed cases, we started antiviral therapy with hydroxychloroquine which may have had a positive effect on postoperative COVID-19-related mortality.

We can also associate the lower than expected mortality of patients with COVID-19 who underwent surgery with the type of surgery and anaesthesia performed. While 52% of the patients who underwent surgery had minor procedures, 31% were operated on using regional anaesthesia techniques. Moreover, although most of the patients had minor surgery, the need for ICU admittance was almost twice as high in the surgical patients compared to those receiving an intervention, and inpatients. Together, these results suggest that secondary trauma, such as surgery, adversely affects the clinical course of COVID-19 patients, even if it does not increase mortality. This secondary impact of surgery was exemplified by an international cohort study of 1128 surgical COVID-19 patients which reported a mortality rate of 16.3% in those who had minor surgery, compared with 26.9% in those who underwent major surgery [10].

According to a Chinese study, 3.8% of confirmed over 70 000 COVID-19 cases were healthcare workers, and only five resulted in death [23]. In our hospitals, 4.3% of confirmed cases were frontline healthcare workers. These numbers seem quite high, but at the beginning of the pandemic, the shortage and improper use of PPE was a problem. COVID-19 transmission occurred in 142 (2.4%) of 5874 healthcare workers, and there were no deaths. Only 3.3% of the infected frontline healthcare workers were anaesthesiologists, and only three of 73 anaesthesiologists working during the pandemic period who performed all airway interventions were infected with SARS-CoV-2. Overall, the infection and contamination rates of the doctors and nurses working in the ward and emergency department were three times higher than those of healthcare professionals working in the operating room ICU and the intervention areas, which possibly reflects more frequent and correct use of PPE. Indeed, a recent study [24] found that with real-time aerosol detection, both tracheal intubation and extubation procedures generate fewer aerosols than a voluntary cough. These findings may explain why healthcare workers in operating rooms, ICUs and intervention areas where airway interventions were frequently performed were infected less frequently. By contrast, a seroprevalence survey of COVID-19 conducted on 17 971 healthcare workers in Denmark found the highest seroprevalence rate (29.7%) in emergency room workers [25]; these findings support a conclusion that the operating room, ICU and intervention areas are safer in terms of contamination and infection risk.

Our study had some limitations. The SARS-CoV-2 PCR test could not be performed on most of the patients due to the unavailability of test kits, and only symptom screening was performed, so we may not have identified all asymptomatic infected patients. Moreover, some of our patients were young and without comorbidities, and even if they were infected, may have had an asymptomatic pneumonia progression. The low number of surgical and intervention patients infected with SARS-CoV-2 decreases the statistical significance, and constitutes an important limiting factor. Therefore, multi-centre studies with a much higher number of patients are required to confirm the observed trends.

In summary, our data show that patients infected with COVID-19 do not have an increased risk of mortality when undergoing surgery and intervention compared to those who were not. However, while surgical patients with COVID-19 required more frequent ICU follow-up, the need for ICU admittance did not increase in intervention patients for whom mechanical ventilation and endotracheal intubation were not performed. Therefore, in patients infected with COVID-19, interventional measures should be preferred to surgery, where clinically appropriate. We conclude that health systems with sufficient capacity and capability could proceed with essential surgical procedures and interventions according to local safety rules and with the effective use of PPE during the COVID-19 pandemic.

## Supplementary material

For supplementary material accompanying this paper visit http://dx.doi.org/10.1017/S0950268821002119. click here to view supplementary material

## Availability of data and materials

The datasets for the current study are available from the corresponding author on reasonable request.
