# Supplementary material for: Are surgical and non-operating room intervention safe in the COVID-19 pandemic? A retrospective study
Source: Epidemiol Infect. 2021 Sep 16;149:e210. doi: 10.1017/S0950268821002119 (PMC8485005; doi:10.1017/S0950268821002119)
Supplement: Supplementary file 1 [file S0950268821002119sup001.docx]

**Supplementary table**

***Comparison of COVID (-) and COVID (+) Surgical and Intervention Patients***

|  | **COVID (-) Group I**  **(*n* = 1234)** | **Preop COVID (+) Group I**  **(*n* = 39)** | *p*_1_ | **COVID (-) Group II**  **(*n* = 446)** | **Preop COVID (+) Group II**  **(*n* = 28)** | *p*_2_ |
| --- | --- | --- | --- | --- | --- | --- |
| Age, years | 37 (32–48) | 57 (41–66) | ***<0 .001*** | 46 (35–59) | 62 (51–72) | ***0.001*** |
| Male, *n* (%) | 337 (27.3) | 21 (53.9) | ***0.002*** | 178 (39.9) | 15 (53.6) | 0.060 |
| BMI, kg/m^2^ | 25.7 (21.8–29.1) | 26.9 (22.8–30.1) | 0.451 | 26.2 (23.1–29.0) | 26.7 (25.0–28.7) | 0.599 |
| ASA classification > 2, *n* (%) | 813 (65.9) | 30 (76.9) | 0.120 | 323 (70.7) | 20 (80.0) | 0.438 |
| Preoperative comorbidities, *n* (%)  Hypertension  DM  CRF  CAD  Immunosuppressive drugs  COPD  Smoking  CVA | 183 (14.8)  106 (8.6)  24 (2.0)  74 (6.0)  72 (5.8)  24 (2.0)  206 (16.7)  15 (1.2) | 19 (48.7)  5 (12.8)  3 (7.7)  4 (10.3)  9 (23.1)  8 (20.6)  12 (30.8)  3 (7.7) | ***<0 .001***  0.302  0.052  0.416  ***<0 .001***  ***<0 .001***  ***0.028***  ***0.006*** | 126 (28.3)  51 (11.4)  9 (2.0)  58 (13.0)  86 (19.3)  35 (7.8)  87 (19.5)  8 (1.8) | 15 (53.6)  6 (21.4)  3 (10.7)  9 (32.1)  7 (25.0)  7 (25.0)  5 (17.9)  2 (7.1) | ***0.001***  0.107  ***0.013***  ***0.003***  0.383  ***0.002***  1.000  0.157 |
| Preoperative laboratories  WBC  Lymphocyte count  NLCR | 8.6 (6.8–10.9)  1.84 (1.43–2.33)  3.2 (2.0–4.7) | 7.7 (5.9–10.7)  1.30 (0.80–1.90)  5.5 (3.0–13.5) | ***0.018***  ***<0 .001***  ***<0 .001*** | 7.7 (6.0–9.9)  1.72 (1.22–2.32)  2.8 (1.9–4.8) | 7.7 (5.9–10.7)  1.30 (0.80–1.90)  5.4 (2.9–9.4) | 0.911  ***0.006***  ***0.011*** |
| Postoperative laboratories  WBC  Lymphocyte count  NLCR | 11.3 (8.9–13.9)  1.48 (1.03–1.97)  6.0 (3.9–9.3) | 10.0 (7.4–17.0)  1.00 (0.72–1.68)  9.1 (4.2–23.9)**^#,§^** | 0.852  ***0.006***  ***0.01*** | 8.2 (6.4–11.1)  1.42 (0.93–1.91)  3.9 (2.4–8.0) | 7.8 (6.3–11.6)  1.34 (0.86–1.84)  4.6 (2.8–7.7) | 0.878  0.548  0.992 |
| Postoperative COVID (+) patients, *n* (%) | 4 (0.2) | — |  | 2 (0.4) | — |  |
| ICU requirement, *n* (%) | 28 (2.3) | 18 (46.2) | ***<0 .001*** | 9 (2.0) | 5 (20.0) | ***< 0.001*** |
| Mortality, *n* (%) | 0 (0.0) | 1 (2.6) | 0.805 | 0 (0.0) | 1 (3.6) | 0.898 |

**CAD**: coronary arterial disease, **COPD**: chronic obstructive pulmonary disease, **CRF**: chronic renal failure, **CVA**: cerebrovascular accident, **DM**: diabetes mellitus, **Group III**: hospitalized COVID patients, **ICU**: intensive care unit, **NLCR**: neutrophil-lymphocyte count ratio,**Group II**: intervention patients, **Group I**: surgical patients, **WBC**: white blood cell, **^#^**Wilcoxon signed rank exact tests, *p* = 0.005 (preoperative NLCR and postoperative NLCR)
